# Cell cycle activity correlates with increased anti‐tumor immunity in diverse cancers

**DOI:** 10.1002/ctm2.98

**Published:** 2020-06-16

**Authors:** Shanmei Jiang, Yin He, Mengyuan Li, Xiaosheng Wang

**Affiliations:** ^1^ Biomedical Informatics Research Lab School of Basic Medicine and Clinical Pharmacy China Pharmaceutical University Nanjing China; ^2^ Cancer Genomics Research Center School of Basic Medicine and Clinical Pharmacy China Pharmaceutical University Nanjing China; ^3^ Big Data Research Institute China Pharmaceutical University Nanjing China

Dear Editor,

The relationship between cell cycle and tumor immunity remains not fully understood, although a recent study demonstrated that cell cycle inhibition could enhance anti‐tumor immunity.^1^ In this study, we investigated the association between the cell cycle activity (CCA) and anti‐tumor immunity in 10 TCGA cancer cohorts, including BLCA, BRCA, COAD, HNSC, KIRC, LIHC, LUAD, PRAD, THCA, and UVM. We found a significant positive association between CCA and CD8+ T cell infiltration levels in the 10 cancer cohorts and their pan‐cancer (Pearson's correlation test, FDR < 0.05) (Figure 1A). In pan‐cancer and in nine cancer cohorts (except LIHC), CCA positively correlated with immune cytolytic activity. Moreover, the ratios of immune‐stimulatory to immune‐inhibitory signatures (CD8+/CD4+ regulatory T cells, M1/M2 macrophages, and pro‐/anti‐inflammatory cytokines) were significantly higher in the higher‐CCA tumors than in the lower‐CCA tumors in most cancer types (Mann‐Whitney *U* test, one‐sided FDR < 0.05) (Figure 1B). Notably, CCA positively correlated with the expression levels of numerous human leukocyte antigen(HLA) genes in cancer, such as *HLA‐B*, *L*, *DOB*, *DPB2*, *DQA1*, *DQA2*, *DQB2*, *DRA* (Figure 1C). Furthermore, we found that a number of immune‐related pathways were upregulated in higher CCA tumors in at least five cancer types, including the pathways of natural killer cell‐mediated cytotoxicity, primary immunodeficiency, allograft rejection, autoimmune thyroid disease, chemokine signaling, cytokine‐cytokine receptor interaction, graft versus host disease, intestinal immune network for IgA production, systemic lupus erythematosus, T‐cell receptor signaling, Toll‐like receptor signaling, hematopoietic cell lineage, leishmania infection, and NOD‐like receptor signaling (Figure 1D). In addition, we found many cell cycle pathway genes showing a positive expression correlation with anti‐tumor immunity in pan‐cancer and in multiple individual cancer types, such as *CDK1*, *CDK6*, *CDC6*, *CDC7*, and *CDC20* (Figure 1E). Collectively, these results indicate a positive correlation between CCA and anti‐tumor immunity in cancer.

FIGURE 1Association of cell cycle activity with anti‐tumor immune signatures in pan‐cancer and in individual cancer types. **A,** Association of CCA with CD8+ T cell infiltration levels and immune cytolytic activity. Pearson's correlation test R and FDR are shown. CCA, cell cycle activity. It also applies to following figures. **B,** Comparison of the ratios of immune‐stimulatory to immune‐inhibitory signatures between higher‐ and lower‐CCA tumors. The Mann‐Whitney U test one‐sided FDR are indicated. The higher‐ and lower‐CCA tumors are the tumors whose CCA scores lie in the upper and bottom third, respectively. A ratio is the log2‐transformed ratio of the geometric mean expression level of all marker genes of an immune‐stimulatory signature to that of an immune‐inhibitory signature in a tumor sample. **C,** Association of CCA with the expression levels of human leukocyte antigen (HLA) genes. **D,** Immune‐related pathways which are highly enriched in higher‐CCA tumors in at least 5 individual cancer types identified by KEGG. **E,** Association of the expression of cell cycle pathway genes with anti‐tumor immune signatures. Pearson's correlation R values are indicated. FDR: false discovery rate, is the adjusted *P* values computed by the Benjamini and Hochberg method (*, FDR < 0.05; **, FDR < 0.01; ***, FDR < 0.001).

**Figure:**
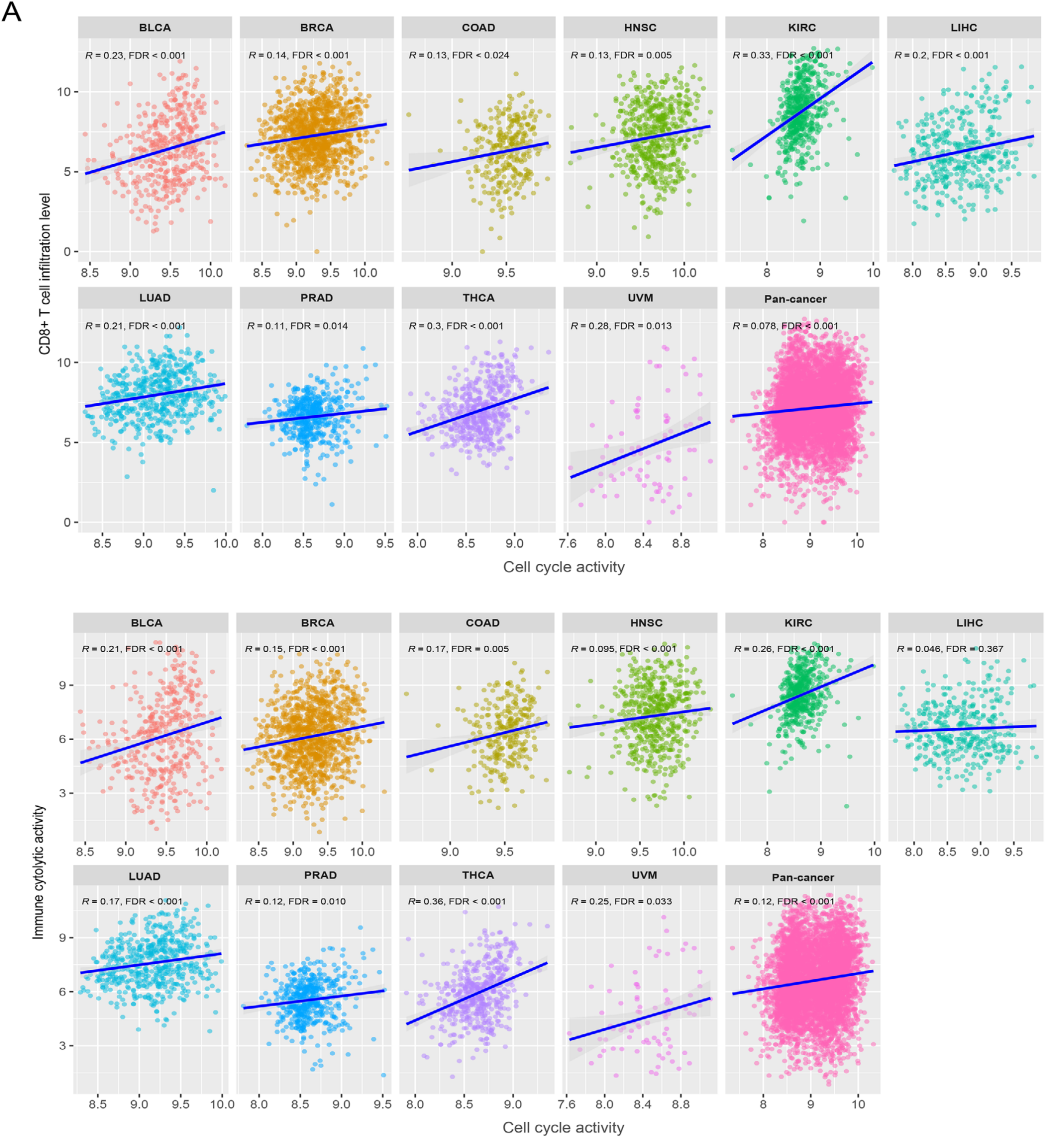

**Figure:**
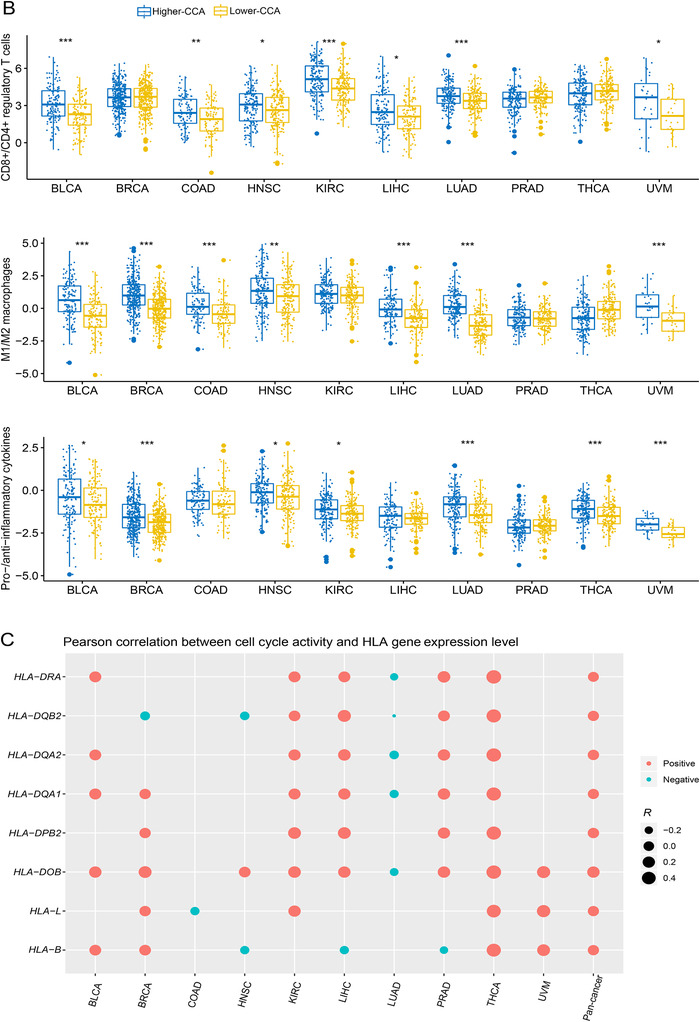

**Figure:**
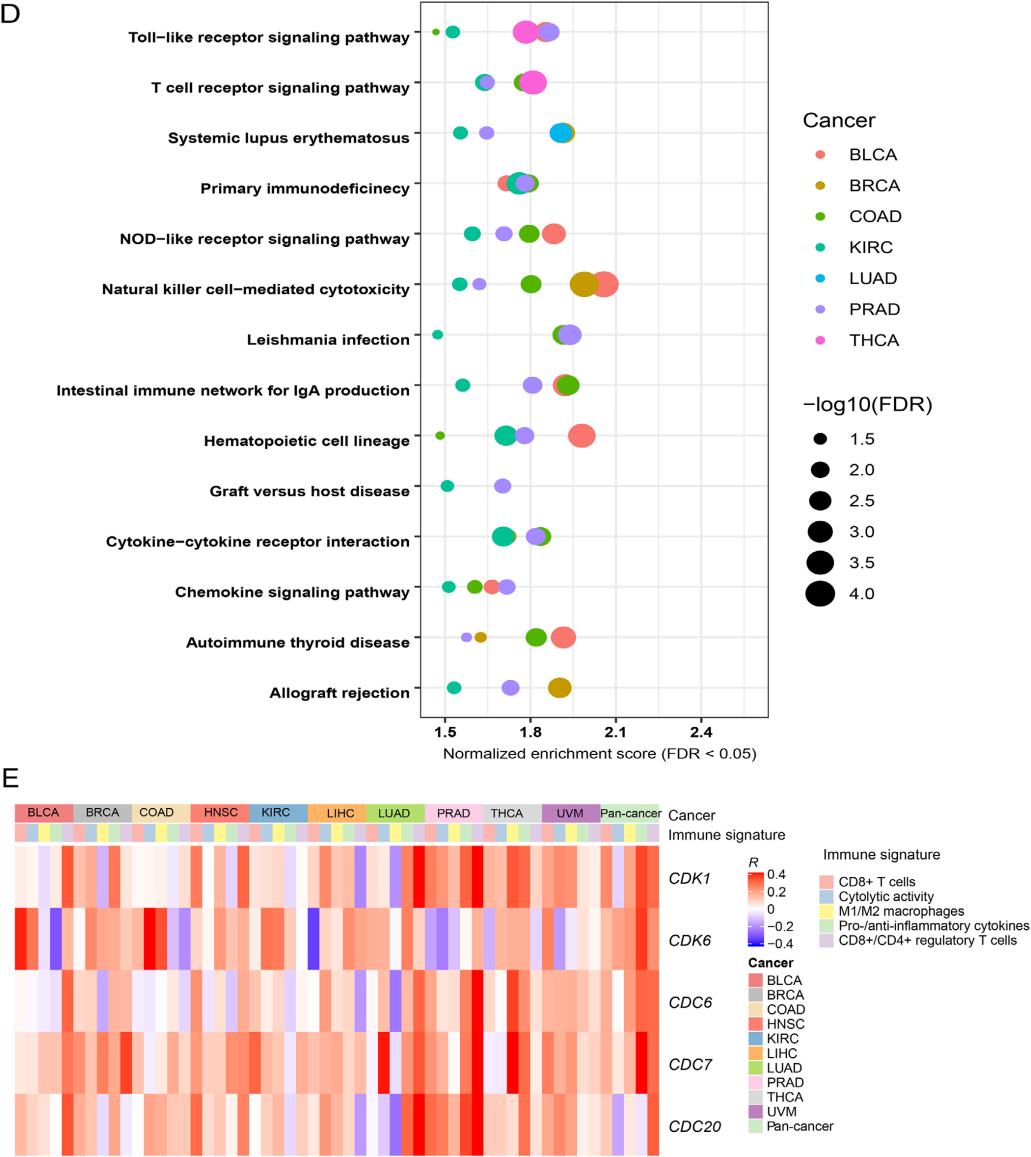

To investigate the predictability of CCA for anti‐tumor immune signatures, we performed logistic regression analyses to estimate the contributions of CCA in predicting CD8+ T‐cell infiltration levels and immune cytolytic activity. Because both tumor mutation burden (TMB)^2^ and tumor aneuploidy level (TAL)^3^, ^4^ have been associated with anti‐tumor immunity, we utilized three predictors (CCA, TMB, and TAL) to predict the tumor samples with high (upper third) versus low (bottom third) immune signature scores in logistic regression models. In pan‐cancer, CCA (β coefficient: β = 0.41, *P *= 4.06 × 10^−6^), TMB (β = 0.39, *P *= 0.001), and TAL (β = ‐0.73, *P *= 1.52 × 10^−13^) displayed significant contributions in predicting CD8+ T‐cell infiltration levels (Figure 2A). The similar results were observed in predicting immune cytolytic activity. These results indicate that anti‐tumor immunity has a significant positive association with CCA and TMB, while it has a significant negative association with TAL. Among the 10 individual cancer types, CCA was a significant positive predictor for CD8+ T‐cell infiltration levels and immune cytolytic activity in seven and six cancer types, respectively (Figure 2A). In these cancer types, although TMB also displayed the potential as a positive predictor, few of the contributions were statistically significant (Figure 2A).In contrast, TAL was a significant negative predictor for CD8+ T‐cell infiltration levels and immune cytolytic activity in six and five cancer types, respectively (Figure 2A). These results suggest that CCA has stronger predictability for anti‐tumor immunity than TMB and TAL in these cancer types.

FIGURE 2Association of cell cycle activity with tumor mutation burden, tumor aneuploidy level, *PD‐L1* expression, immunotherapy response, and DNA damage repair pathways. **A,** Logistic regression analysis of CCA, TMB, and TAL for predicting immune signatures (CD8+ T cell infiltration levels and immune cytolytic activity) in pan‐cancer and in individual cancer types. The tumor status of high (upper third) or low (bottom third) immune signature scores were predicted. TMB is the total somatic mutation count in tumor and tumor aneuploidy level is the ploidy score calculated by ABSOLUTE. β coefficients and *P* values for each predictor are shown. CCA, cell cycle activity. TMB, tumor mutation burden. TAL, tumor aneuploidy level. **B,** Association of CCA with *PD‐L1* expression levels. Pearson's correlation test R and *P* values (FDR) are indicated. **C,** Association of the expression levels of cell cycle genes with *PD‐L1*. Pearson's correlation test R values are indicated. **D,** Kaplan‐Meier survival curves showing that higher‐CCA cancer patients have better survival trends than lower‐CCA patients in Snyder (urothelial cancer) cohort receiving anti‐PD‐L1 therapy. **E,** The elevated expression of *CDK7* is associated with increased anti‐PD‐L1 response and better survival prognosis in Snyder cohort. **F,** Kaplan‐Meier survival curves showing that the mutations of many cell cycle pathway genes are associated with better overall survival in Samstein (pan‐cancer) cohort receiving anti‐PD‐1/PD‐L1/CTLA‐4 therapy (log‐rank test, *P* < 0.05). **G,** Significant positive correlations between CCA and TMB in pan‐cancer and in five cancer types. Spearman's correlation test ρ and adjusted *P* values (FDR) are shown. **H,** Association of CCA with the activity of DNA damage repair pathways. Pearson's correlation test R values are shown. **I,** The ratios of the mean expression levels of *CD8A* to *PD‐L1* are significantly lower in higher‐CCA (upper third) than in lower‐CCA (lower third) tumors in pan‐cancer and in four cancer types. Student’s t test adjusted *P* values are shown (*, FDR < 0.05; **, FDR < 0.01; ***, FDR < 0.001).

**Figure:**
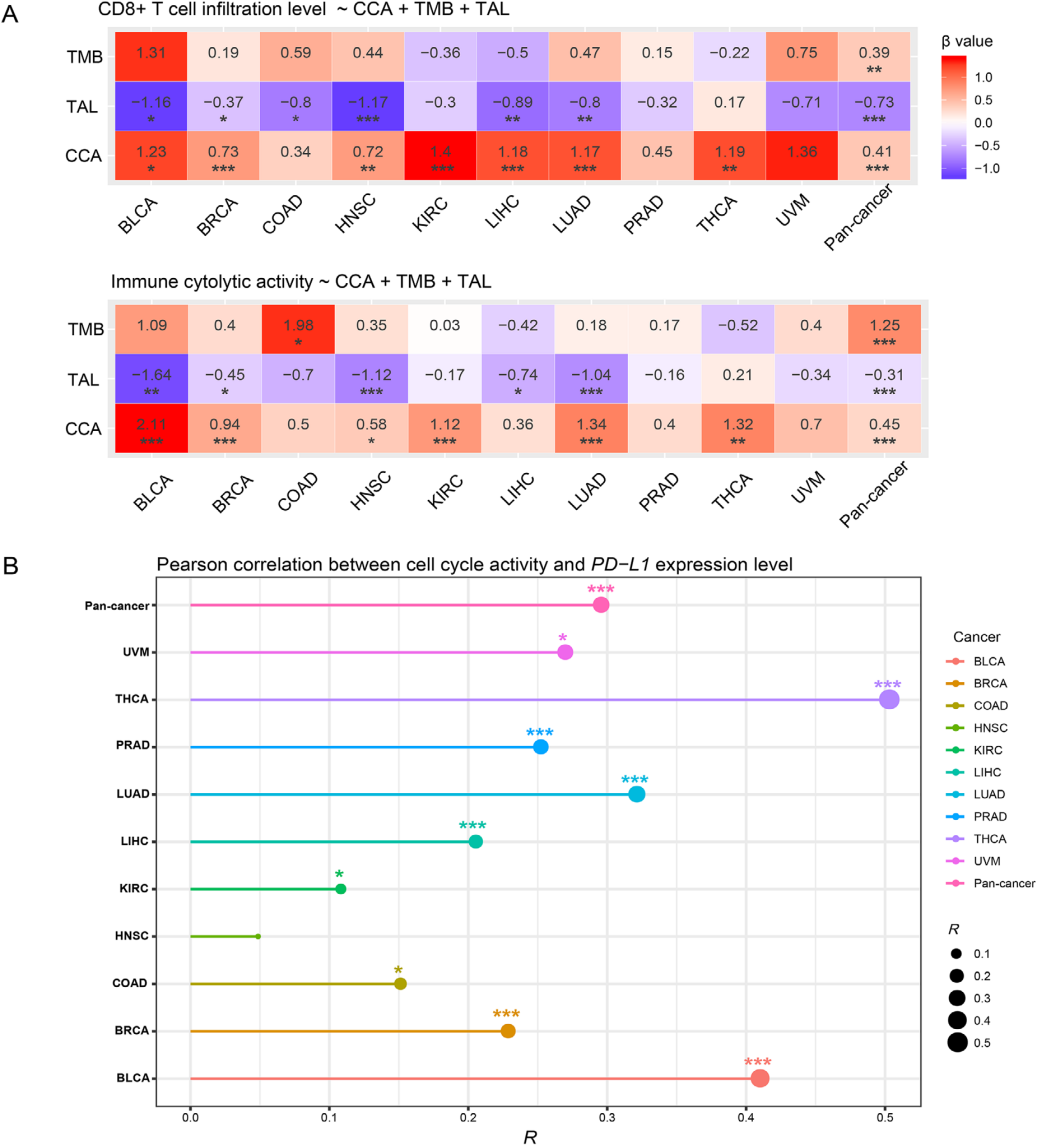

**Figure:**
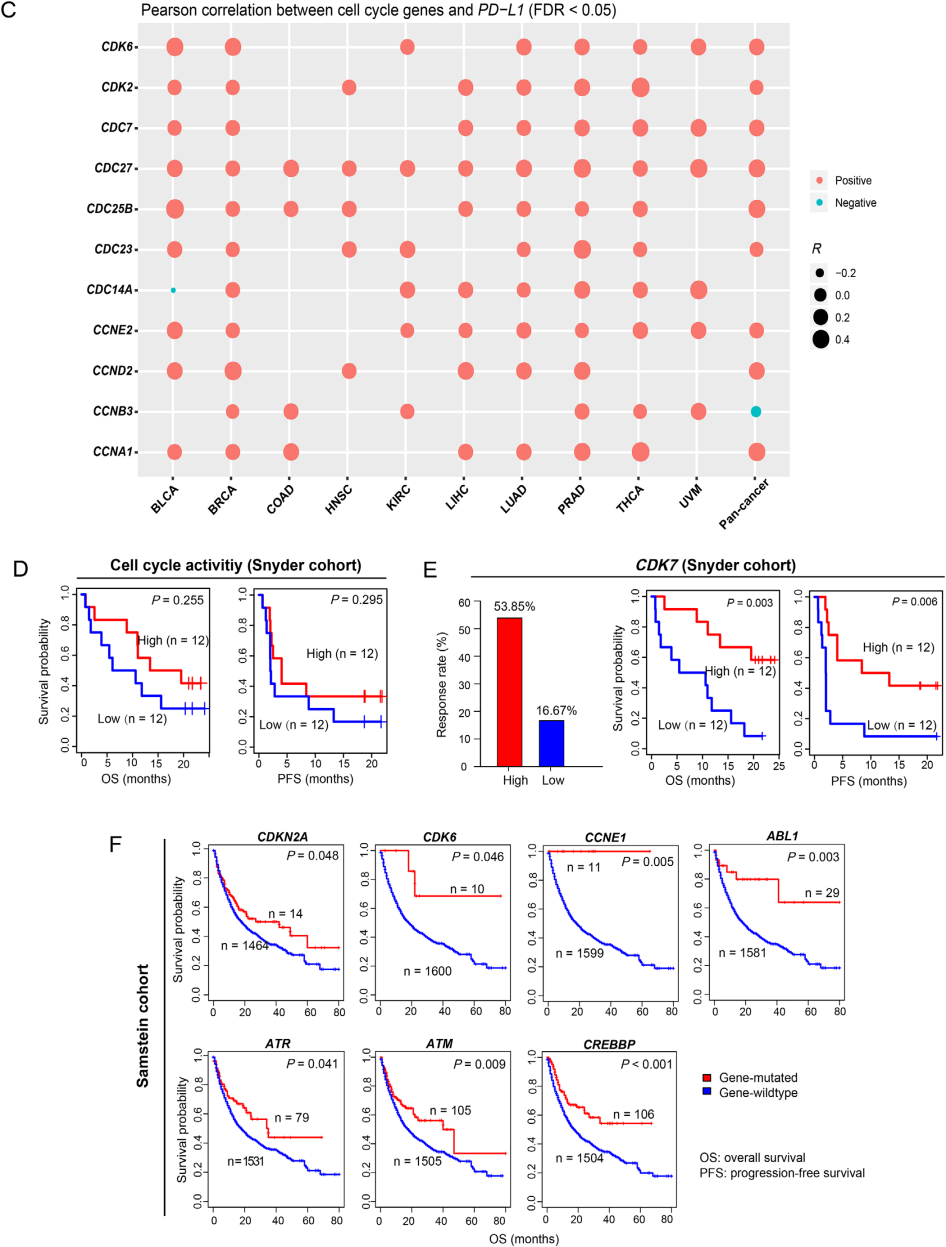

**Figure:**
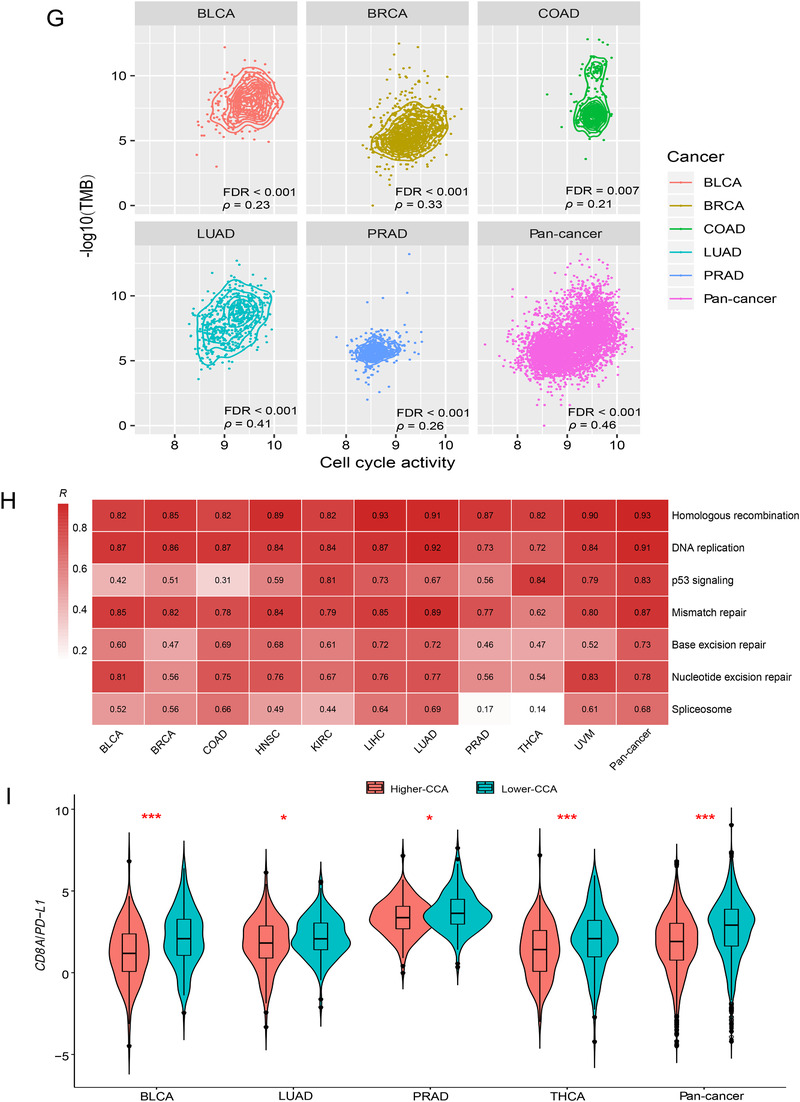

Interestingly, we observed significant positive correlations between *PD‐L1* expression levels and CCA in pan‐cancer and in nine individual cancer types (Figure 2B). Moreover, 11 cell cycle pathway genes showed significant positive expression correlations with *PD‐L1* in at least five cancer types (Figure 2C). Since the PD‐L1 expression is a predictive biomarker for the active response to anti‐PD‐1/PD‐L1 immunotherapy,^5^ we anticipated that high‐CCA cancer patients would have a more favorable response to immunotherapy. Indeed, in a urothelial cancer cohort^6^ receiving anti‐PD‐L1 therapy, higher CCA cancer patients showed more favorable overall and disease‐free survival trends than lower CCA patients (Figure 2D). Moreover, the upregulation of *CDK7*, a member of the cyclin‐dependent protein kinase family, was associated with a better survival in this cancer cohort (Figure 2E). The positive correlation between *CDK7* expression and survival prognosis could be attributed to the elevated anti‐PD‐L1 response rate in the cancers with higher *CDK7* expression levels (Figure 2E). Moreover, in another cancer cohort receiving anti‐PD‐1/PD‐L1/CTLA‐4 therapy,^7^ we found a number of cell cycle pathway genes whose mutations were associated with better overall survival (Figure 2F).

One possible reason why CCA can promote anti‐tumor immunity could be that CCA enhances TMB.^8^ Indeed, we observed significant positive correlations between CCA and TMB in pan‐cancer and in five cancer types (Figure 2G). Moreover, we found several DNA damage repair‐associated pathways, which were highly enriched in higher CCA tumors in at least five cancer types, including homologous recombination, DNA replication, p53 signaling, mismatch repair, base excision repair, nucleotide excision repair, and spliceosome. CCA displayed a strong positive correlation with the activity of these pathways in pan‐cancer and in most individual cancer types (Figure 2H), indicating that the positive association between CCA and anti‐tumor immunity is DNA damage repair mediated.

The tumors with higher CCA likely have more unfavorable clinical phenotypes, although they were associated with increased anti‐tumor immunity. A possible explanation could be that CCA promotes tumor immunosuppressive signatures (such as PD‐L1) as well, thereby counteracting the effect of elevated anti‐tumor immunity. In fact, we found that the ratios of the mean expression levels of *CD8A* (CD8+ T cell marker) to *PD‐L1*were significantly lower in higher CCA tumors than in lower CCA tumors in pan‐cancer and in four cancer types (Figure 2I). This indicates that CCA has a stronger positive correlation with the tumor immune‐suppressive signature (PD‐L1) than with the anti‐tumor immune signature (CD8+ T cells) in these cancer cohorts.

In conclusion, CCA has significant positive associations with anti‐tumor immunity in diverse cancer types. The combination of cell cycle inhibitors and immunotherapy should be cautious since CCA is positively associated with the response to immunotherapy.

## CONFLICT OF INTEREST

The authors declare no conflict of interest.

## AUTHOR CONTRIBUTIONS

Shanmei Jiang performed data analyses and helped prepare for the manuscript. Yin He performed data analyses and helped prepare for the manuscript. Mengyuan Li performed data analyses. Xiaosheng Wang conceived this study, designed analysis strategies, and wrote the manuscript. All the authors read and approved the final manuscript.

## Supporting information

Supporting Information

## Data Availability

All the data and materials are available upon reasonable request from the authors.
